# CTX-M-127 with I176F mutations found in bacteria isolates from Bangladeshi circulating banknotes

**DOI:** 10.1038/s41598-024-56207-x

**Published:** 2024-03-11

**Authors:** Md. Zannat Ali, Sankaranarayanan Srinivasan, Selina Akter

**Affiliations:** 1 Department of Microbiology, Jashore University of Science and Technology, Jashore, Bangladesh; 2 Department of Biotechnology, Indian Institute of Technology Madras, Chennai, India

**Keywords:** CTX-M-127, CTX-M-15, Banknotes, Antibiotic resistance, Extended-spectrum beta-lactamase, Mutation, Computational biology and bioinformatics, Microbiology, Natural hazards

## Abstract

Extended-spectrum beta-lactamase (ESBL)-producing organisms are widely recognized as clinically relevant causes of difficult-to-treat infections. CTX-M has formed a rapidly growing family distributed worldwide among a wide range of clinical bacteria, particularly members of *Enterobacteriaceae*. Circulating banknotes, exchanged daily among people, pose a potential vehicle for transmitting multidrug resistance. We screened for ESBL-carrying bacteria in the present study and reported CTX-M mutations in Bangladesh's banknotes. We sequenced the genes and performed homology modeling using the Swiss model with CTX-M-15 (4HBT) as a template. Then, we performed molecular docking of mecillinam with the template and the generated model using Autodock 4.2 (Release 4.2.6). After docking, we visually inspected the complexes built using Autodock tools for polar contacts and pi-pi interactions in PyMOL 2.5.4. Our partially sequenced *bla*_CTX-M_ was related to *bla*_CTX-M-10_ and *bla*_CTX-M-15_. We observed multiple single-nucleotide substitution mutations, i.e., G613T (silent mutation), A626T (I176F), and A503G (N135D). Homology modeling showed high similarity when the model was superimposed over the template. The orientation of Asn (135) in the template and Asp (135) in the model does not show a significant difference. Likewise, Ile (176) in the template and Phe (176) in the model offer the same orientation. Our generated model could bind to Lys237, Ser240, and Asp135 residues with the lowest binding energy on docking. Our predicted binding of the mecillinam to the mutated D-135 residue in the model indicates contributions and supports previous reports proposing CTX-M-15 to CTX-M-127 mutational conversion on the mecillinum resistance phenotype.

## Introduction

The most common cause of bacterial resistance to β-lactam antibiotics is the production of β-lactamases. Extended Spectrum Beta-lactamases (ESBLs) have co-evolved and emerged against each of the new classes of β-lactams introduced and caused resistance^1^. ESBL-producing organisms, first identified in Germany in 1983, are now widely recognized as clinically relevant causes of infections in the community^2^. It was related to the production of a variant of the SHV-1 enzyme, followed by the description of TEM-1 and TEM-2 enzyme variants in France with hydrolytic properties similar to SHV-1 derivatives. They were named extended-spectrum β-lactamases (ESBLs) in 1989^3^. At the same time, a new family of ESBL, called the CTX-M, was detected in humans in Germany on a clinical cefotaxime-resistant *Escherichia coli* strain, which produced a non-TEM, non-SHV ESBL. It was designated CTX-M-1, referring to its hydrolytic activity against cefotaxime^4^. Following this, the prevalence of ESBL harboring bacteria like *E. coli*, *Salmonella* sp., and different classes of *Enterobacteriacea*e was reported in France^5^, Japan^6^, Argentina^7^, and Poland^8^, though called with different names. Since then, the CTX-M enzymes have formed a rapidly growing family of ESBLs distributed over vast geographic areas and among a wide range of clinical bacteria, in particular, members of the family of *Enterobacteriaceae*^9^. Currently, the CTX-M family comprises at least 265 enzymes (in the Beta-lactamase database, BLDB, assessed on September 2023^10^).

Multidrug-resistant bacteria spread across communities through various modes. In the early 1900s, scientists theorized that money transmission was associated with disease transmission^11^. Modern scientific techniques have confirmed these theories and shown that viable pathogenic organisms can be isolated on the surfaces of paper and coin currencies^12^. Paper banknotes, handled by a large number of people, increase the possibility of acting as a vehicle for the transmission of pathogenic microorganisms^13^.

Paper banknotes with lower denominations commonly carry more bacterial contamination due to circulation frequency^14^. Currency papers from India, Ghana, Bangladesh, and Iraq were found to carry pathogenic or potentially pathogenic bacteria^15^. Bank notes having ESBL producing *E. coli* and *Klebsiella* spp., *Pseudomonas* spp. contribute to transmitting these multidrug-resistant microorganisms in the community^16^. Taxonomical distribution of the microbiome could provide insights into the bacteria carrying antibiotic-resistant genes. Using 16S ribosomal RNA-based shotgun metagenomics approaches, Kohli et al.^17^ identified 78 antibiotic resistance genes across various bacteria. Among those, about 18 genes, including class A beta-lactamases, were found in all the samples.

Bangladesh has approved a National Action Plan (NAP) for containing antimicrobial resistance (AMR) in alignment with the WHO GAP guidelines. Despite the Government's strict measures preventing antibiotic overuse and misuse, antibiotics are still available over the counter, especially in remote rural areas and for agricultural use in animal husbandry and poultry industries^18^. Inappropriate use of antibiotics causes a high prevalence of resistance to pathogens in Bangladesh. The presence of ESBL-producing organisms was indicated to be responsible for resistance to beta-lactams^19^. We screened for ESBL-carrying bacteria in Bangladesh's banknotes in the present study. We reported CTX-M-127 for the second time with I176F mutation, suggesting a well-established prevalence of the novel CTX-M mutants in this region. CTX-M-127 confers resistance to mecillinam^20,21^. However, the mechanism of resistance has yet to be established. Here, we probed further into the resistance by checking if the mutated D135 interacts with mecillinam in silico and predicted that this interaction indeed occurs. For this, template-based modeling such as the Swiss model^22^ could be the model of choice with molecular docking using Autodock-4.2 (Release 4.2.6)^23^. The Swiss model generates models for proteins without structure by searching for templates in its repository, aligning the sequence with templates from the repository or using the user-defined template, extracting the structural features, and finally, building as well as evaluating the model. This tool is a better choice if the similarity between the template and the protein to be modeled is very high, as in our case.

## Experimental procedure

### Sample collection, enrichment, and bacterial isolation

We have collected Bangladeshi paper banknotes of varying denominations (BDT 2, 5, 10, 20, 50, and 100) from different locations of the Jashore district between September 2022 and June 2023. Samples were collected from people of different occupations (randomly selected rickshaw pullers, fish and vegetable retailers, bus ticket collectors, grocery shopkeepers, beggars, and students) as an exchange of purchase goods or services and transported to the laboratory within 2–3 h in a sterile sample collection bag without any transport media.

We dipped each sample in 10 ml of autoclaved phosphate buffer saline (PBS) containing 1 g of glass beads and agitated for 30 min at 80 rpm in an arbitrary shaking incubator at 30 °C. We removed the banknotes, washed them in tap water, and air-dried them to use further for exchange. We used 1 ml of PBS wash spent as a sample to inoculate 5 ml of nutrient broth (Oxoid, UK) containing antibiotics for enrichment. We used combinations of 3rd generation cephalosporins (Ceftriaxone, Cefotaxime, and Ceftazidime at a concentration of 8 µg/ml each) and/or a carbapenem (Meropenem at 4.0 µg/ml). We incubated the enrichment broth at 37 °C for 18 h with continuous shaking at 120 rpm. We inspected the nutrient broth culture cultures for visible turbidity and streaked on MacConkey agar (MCA) if bacterial growth was visible. After overnight incubation at 37 °C, we picked discrete pure colonies and sub-cultured on the corresponding agar media. We screened isolated colonies based on the colony morphology and pigmentation on selective media and identified by biochemical tests (citrate utilization Test, methyl red and Voges-Proskauer (MR-VP) test, Triple sugar iron agar test, indole production, lactose fermentation, H_2_S fermentation tests), motility, bacterial morphology, arrangements and Gram reaction.

### Antibiotic susceptibility test

We have carried out the Kirby-Bauer Disk diffusion method^24^ and interpreted it according to the method described by the National Committee on Clinical Laboratory Standards (NCCLS) (1999) guidelines. In short, we transferred colonies from pure overnight culture into 2 ml of normal saline and adjusted the concentration to 0.5 McFarland standards. We spread the inoculum on the surface of the Mueller–Hinton agar (Oxoid, UK) plate with a sterile swab and let that air dry. We gently pressed the commercial discs of selected antibiotics (Ceftriaxone, Cefotaxime, Ceftazidime, and Meropenem) on the plate at a distance of 20 mm apart. We measured the diameter of bacterial growth inhibition around each disc after incubation at 37 °C for 18–20 h. We also carried out the Combined disc test^25^ for phenotypic differentiation between the presence of *Klebsiella pneumonia* carbapenemase (KPC) and Metallo beta-lactamase (MBL) enzyme using commercially prepared discs containing 10.0 µg Meropenem with/without Bronic acid (600 µg/disc) and/or EDTA (730 µg/disc).

### Identification of beta-lactamase genes

To detect the beta-lactamase genes in the isolated and identified pure bacterial isolates, we inoculated 10 ml of nutrient broth media and incubated overnight at 37 °C. Bacterial cells were harvested by centrifugation at 12,000*g*, and the pellet was washed once with sterile PBS and used for DNA extraction. We used the alkaline lysis method followed by phenol: chloroform: isoamyl alcohol extraction protocol to extract bacterial DNA, which was used as a template for Polymerase Chain Reaction (PCR). For PCR amplification of beta-lactamase genes, we used 2X PCR Master Mix (DreamTaq Green PCR Master Mix, Thermo Scientific, USA). To prepare 10 µl reaction volume, we mixed 5µl master mix, 3µl Nuclease-free water, 0.5µl each of forward and reverse primers (10 pmol), and 1.0 µl of DNA template at last after a short spin. We set the thermocycler (Bio-Rad) conditions for 35 cycles of the regime: denaturation at 95 °C for 1 min, annealing at different temperatures (CTX-M-1: 62 °C; CTX-M-2: 55 °C; CTX-M-8: 60 °C; CTX-M-9: 55 °C; CTX-M-10: 60 °C, CTX-M-14: 52 °C; TEM: 56 °C; KPC: 60 °C; and NDM: 60 °C) and extension at 72 °C with an initial denaturation at 95 °C for 5 min and final extension at 72 °C for 7 min. We provided the primer sequences in the supplement (Table S1). We prepared 1.5% agarose gel using 1% ethidium bromide (EtBr) solution (at 6 µl/100 ml gel concentration) for electrophoretic separation of amplified PCR products in TAE buffer. We visualized the DNA amplicon as bands in agarose gel after electrophoresis using a UV illuminator (BioRad). We used a molecular weight marker to measure the amplicon lengths and Gel Documentation software (Gel Doc Systems, Thermo Fisher Scientific) to capture photographs.

### Sequencing of PCR amplicon

We used the same amplified PCR products as a template for sequencing using the dideoxy nucleotide chain termination method^26^ using SeqStudio™ Genetic Analyzer (Applied Biosystems, USA) after purifying the PCR products with the ExoSAP-IT™ PCR product cleanup reagent as per manufacturer instruction (Thermo Fisher Scientific, USA). We used BigDye Terminator v3.1 cycle sequencing ready-to-use reaction kit (Thermo Fisher Scientific). We set up the cycle sequencing PCR condition according to the kit protocol. We submitted the DNA sequences and translated peptide sequences (using Open Reading Frame Finder (https://www.ncbi.nlm.nih.gov/orffinder/) through BankIT (https://www.ncbi.nlm.nih.gov/WebSub/) and Accession IDs to the submitted sequences as an archetype are available in the NCBI database (Table 1).

**Table 1:** List of sequences performed in the study with the feature of naturally acquiring single nucleotide substitution mutations in blaCTX-M gene and effect on beta-lactamases.

Sample ID	Sequence Accession number	Single nucleotide substitution mutation	Amino acid substitution	Feature
M50T2-a MAC (34f)	OR578821	No mutation	–	*bla*_CTX-M-10_ or feature of *bla*_CTX-M-15_
M10T8-c MAC (37f)	OR578822	No mutation	–	*bla*_CTX-M-10_ or feature of *bla*_CTX-M-15_
C10T8-c MAC (51f)	OR578823	A503G, G613T^1^, and A626T	N135D and I176F	*bla*_CTX-M-127_ with unique mutations
C10T7-b MAC (53f)	OR578824	G613T^1^, and A626T	I176F	*bla*_CTX-M-10_ or feature of *bla*_CTX-M-15_ with unique mutations
C50T2-b MAC (57f)	OR578825	G613T^1^, and A626T	I176F	*bla*_CTX-M-10_ or feature of *bla*_CTX-M-15_ with unique mutations

^1^Silent mutation.

### Mutation analysis

We performed further bioinformatics analyses considering CTX-M sequences in Beta-lactamase database, BLDB (http://bldb.eu/BLDB.php?prot=A#CTX-M) as the reference sequences using Molecular Evolutionary Genetics Analysis (MEGA X) software^27^. At DNA and peptide levels, we utilized MEGA to identify mutations, including substitutions, insertions, deletions, and frameshift mutations, by comparing sequences to BLDB references for all CTX-M beta-lactamases. In addition to mutation analysis, we constructed phylogenetic trees to visualize the evolutionary relationships among the sequences and estimated ancestral states to gain insights into the history of mutations.

### Homology modeling of the novel CTX-M protein

The CTX-M gene we found was similar to CTX-M-127. This enzyme also harbors the same mutation, N135 (found in CTX-M-15) to D135. Since there was no crystal structure available for CTX-M-127, we used the structure of CTX-M-15 (PDB ID- 4HBT) as the template for homology modeling using the Swiss model's 'use template' module (https://swissmodel.expasy.org/interactive#structure)^22^ because it differs from the template CTX-M-15 (PDB: 4HBT) only by two residues from ours. We verified the RMSD value of the generated model and the template (PDB-4HBT) using the structure assessment tool of the Swiss model (https://swissmodel.expasy.org/assess). The RMSD value generated by the structure assessment tool was compared with the RMSD value generated using PyMol's ‘align protein1, protein2 cycles = 0 transform = 0’ command after superimposing the modeled structure with the template^28^. We accepted the model structure for docking purposes if both tools' RMSD values were less than 0.5 Å.

### Molecular docking

Molecular docking was performed with autodock 4.2 (Release 4.2.6) according to Morris et al.^23^ and Khan et al.^29^ using Lamarckian Genetic Algorithm. For docking, we used CTX-M-15 (PDB: 4HBT) as a positive control to compare the binding of mecillinam with the homology model. Before carrying out a docking experiment, the binding site or the residues involved in interaction with the protein’s cognate ligands must be found out. This information can be extracted from the structures that are generated by co-crystallizing the protein with their cognate ligands. Prior to docking, we identified the avibactam (NXL104) interacting residues using the 'find polar contacts' feature of pymol in the crystal structure (PDB: 4HBU) of CTX-M-15^30^ and used these residues as reference for checking the binding of mecillinam to CTX-M-15 (PDB: 4HBT) and the model. The grid dimensions were specified accordingly to cover these residues. Then, the protein and the ligand mecillinam were converted to PDBQT (Protein Data Bank, Partial Charge (Q), & Atom Type (T)) format files using autodock tools. The grid dimensions specified were spacing 0.375, npts x-48 y-48, z-40, and center grid box, x-center: − 4.951, y-center: − 1.598, and z-center: 14.768. For docking using the genetic algorithm, we used the following parameters viz. 300 initial populations, 25,000,000 evaluations, 27,000 generations, and ten independent LGA runs. Post docking, the Autodock gave ten complexes. Among these, we selected the complex with the lowest free energy. The selected complex was built using Autodock tools and then checked visually for polar contacts and pi-pi interactions between mecillinam and the mutant protein in PyMOL 2.5.4.

## Results and discussion

ESBLs have been predominant beta-lactamases that mediate gram-negative bacteria resistance to new broad-spectrum beta-lactam antibiotics. *CTX-M* types are the major phenotypes of domestic ESBLs, which have been reported to be prevalent worldwide^31^. We collected 60 samples across the Jashore district of Bangladesh to isolate antibiotics-resistant bacteria from the banknotes. Using three different enrichments, we ultimately recovered 42 Gram-negative pure bacterial isolates (54 with Ceftriaxone + Cefotaxime + Ceftazidime enrichment, 21 with Meropenem enrichment, and 15 isolates from Ceftriaxone + Cefotaxime + Ceftazidime + Meropenem). Among the bacterial isolates, *Escherichia coli* was the most prevalent found (n = 16), followed by *Pseudomonas* spp. (n = 14), *Acinetobacter* spp. (n = 8), and *Klebsiella* spp. (n = 4). We assessed the antibiotic susceptibility of all these 42 isolates using the Kirby-Bauer Disk diffusion method^24^ and interpreted it according to the method described by the National Committee on Clinical Laboratory Standards (NCCLS) (1999) guidelines. All these 42 isolates were resistant to Ceftriaxone (30 µg), Cefotaxime (30 µg), Cefoperazone (30 µg), Ceftazidime (30 µg), Meropenem (10 µg), Imipenem (10 µg), Aztreonam (30 µg). Detailed results for the zone of bacterial growth inhibition have been given in the supplement (Table S2).

### Identification of beta-lactamase gene by Polymerase Chain Reaction (PCR)

We used nine primer pairs for the amplification of *bla*_CTX-M-1,_ *bla*_CTX-M-2,_ *bla*_CTX-M-8,_ *bla*_CTX-M-9,_ *bla*_CTX-M-10,_ *bla*_CTX-M-14,_ *bla*_TEM,_ *bla*_KPC,_ and *bla*_NDM_ genes. Among the 42 Gram-negative bacterial isolates recovered from banknotes, 25 isolates harbored at least one of the tested beta-lactamase genes. We found 15 isolates PCR positive for *bla*_CTX-M-1_ gene, three isolates for *bla*_CTX-M-8_ gene, 18 isolates for *bla*_CTX-M-10_ gene, 13 isolates for *bla*_CTX-M-14_ gene, two isolates for *bla*_KPC_ gene, 18 isolates for *bla*_TEM_ gene, and nine isolates for *bla*_NDM_ gene. We did not find any isolates PCR positive for *bla*_CTX-M-2_ and *bla*_CTX-M-9_ gene. Figures for representative agarose gels are provided in the supplement (Fig. S1). We observed that some isolates carried multiple beta-lactamase genes simultaneously. Detailed results on beta-lactamase genes present in the isolates are listed in the supplement (Table S3). This article will discuss five randomly selected CTX-M-10 beta-lactamase-producing isolates and their gene sequences.

### Sequence analysis found single-nucleotide substitution mutations

**Figure 1:**
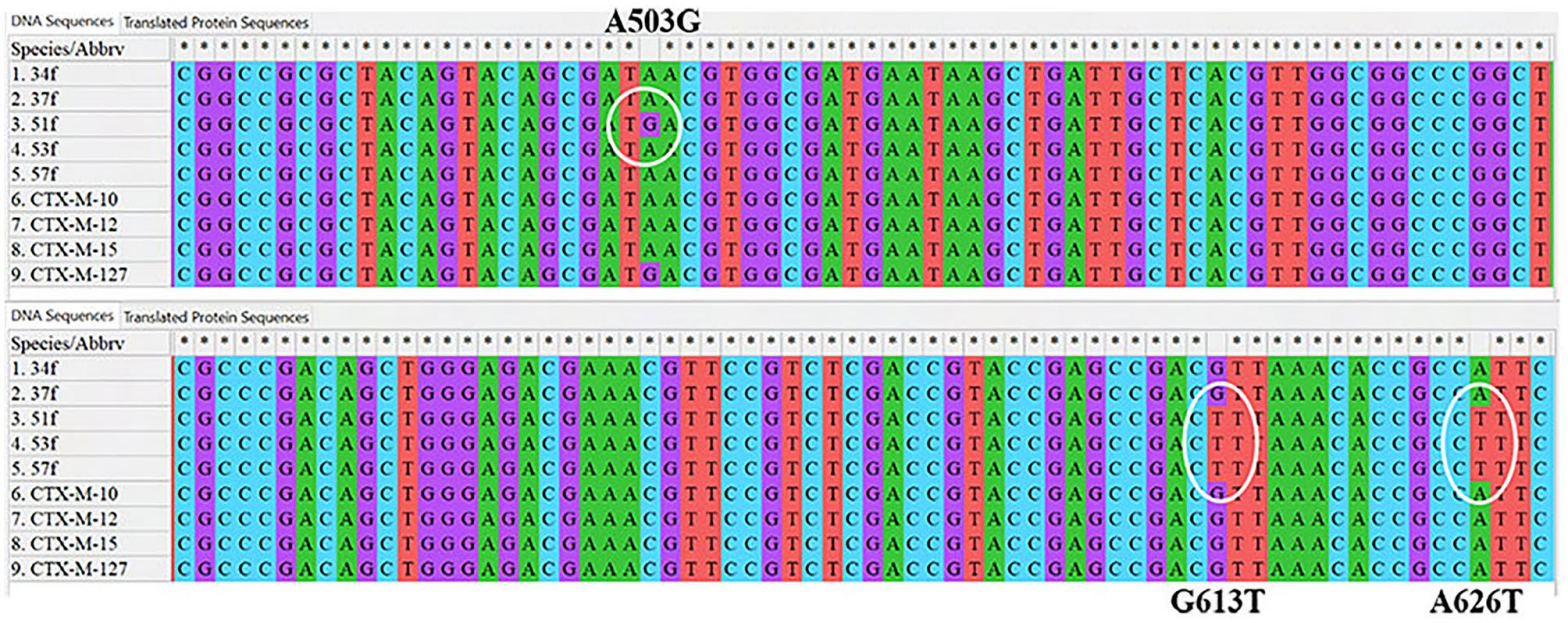
Single nucleotide substitution mutations are shown in sequences aligned with closely related CTX-M beta-lactamase gene (blaCTX-M-10, blaCTX-M-15 and blaCTX-M-127) reference sequences.

**Figure 2:**
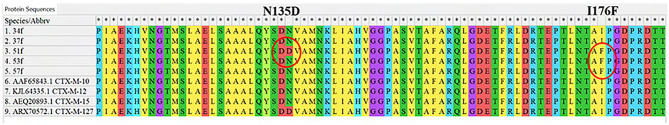
Mutational changes in amino acids by single nucleotide substitution mutations are shown in the translated peptide sequences aligned with closely related CTX-M beta-lactamase enzymes CTX-M-10, CTX-M-15, and CTX-M-127 sequences.

Purified PCR products (amplicon size 524 bp), amplified using primer pairs (Table S1) that target *bla*_CTX-M-10_ and similar CTX-M type beta-lactamase gene lineages, are selected for DNA sequencing. We randomly chose five isolates designated as M50T2-a MAC, M10T8-c MAC, C10T8-c MAC, C10T7-b MAC, and C50T2-b MAC. Biochemical tests identified these isolates as *E. coli* (first three in the list), *Pseudomonas* sp. (fourth in the list), and *Acinetobacter* sp. (last in the list). We observed the partial sequence of *bla*_CTX-M-10_ gene family and identified multiple single-nucleotide substitution mutations among three of five sequences (GenBank Accession number OR578821-OR578825). The partial sequence we received from isolate M50T2-a MAC (34f) and M10T8-c MAC (37f) did not acquire any mutation and aligned identically to that of reference *bla*_CTX-M-10_ or related gene *bla*_CTX-M-15_. Partial sequences of *bla*_CTX-M_ belong to isolates C10T8-c MAC (51f), C10T7-b MAC (53f), and C50T2-b MAC (57f) acquired single nucleotide substitution naturally. We aligned our sequences with reference *bla*_CTX-M_ gene sequences retrieved from BLDB, analyzed the phylogenetic relationship (Fig. S2) and have shown single nucleotide substitution mutations in alignment with closely related CTX-M beta-lactamase gene (*bla*_CTX-M-10_, *bla*_CTX-M-15_ and *bla*_CTX-M-127_) sequences (Fig. 1). We have provided the list of mutations and their effect on amino acid sequences in Table 1. We specifically want to mention the Adenine (A) substituted with Guanine (G) at the 503 base position of the CTX-M-15 beta-lactamase gene brings out an N135D mutational change (Fig. 2). N135D mutation has been reported once early in a human case study^28^ and renamed as *bla*_*CTX‐M‐127*_ gene. This particular mutation was also identified as a common in vitro^29^ mutation after mecillinam selective pressure on a *bla*_*CTX‐M‐15*_ positive isolate. It was associated with resistance to mecillinam and increased susceptibility to ceftazidime. To our knowledge, bla_CTX‐M‐127_ is being identified for the second time as a naturally selected mutant after a single case in Danish surveillance^28^. Concerning all three base substitution mutations, we tried to stipulate the effect on beta-lactamase enzyme activity in silico. G613T is a silent mutation, and we decided not to discuss it further. Nielsen, Hansen et al.^28^ reported that A503G (N135D) mutational conversion from *bla*_CTX-M-15_ to *bla*_CTX-M-127_ correlated mecillinam resistance phenotype during pivmecillinam treatment of a patient, though mechanism yet to be explained. In our study, we looked into the exact mechanism and used mecillinam as a ligand for our docking analysis to postulate the resistance. We also look into our unique mutation A626T (I176F) in homology modeling and docking parameters.

### Mecillinam binds to the mutated D135 in the model, indicating a possible contribution to resistance

**Figure 3:**
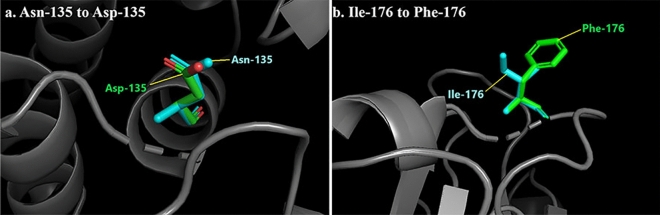
Superimposition of the model and the template CTX-M-15 (PDB: 4HBT). The model/mutant's residues are in green, while the template CTX-M-15's residues are in cyan color.

**Figure 4:**
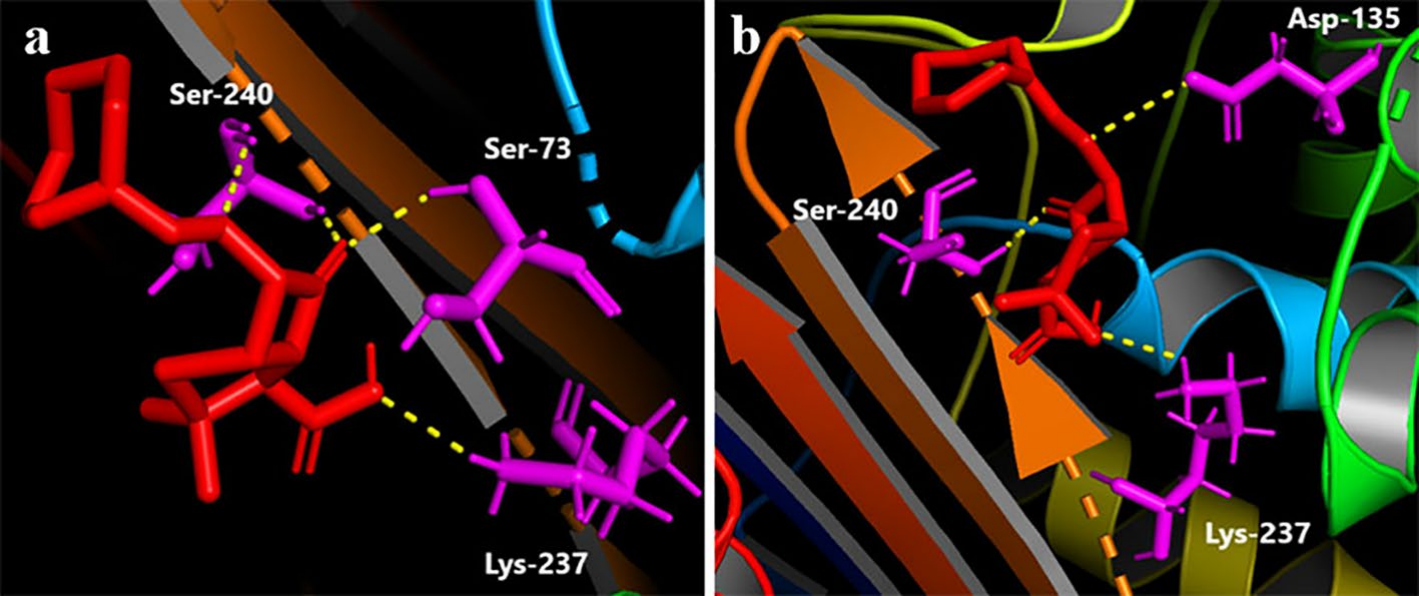
Binding of mecillinam to wild-type CTX-M and modeled protein harboring the mutations N135-D135 and I176-F176. The molecule in red is mecillinam, and the molecules in magenta are the amino acid residues that mecillinam interacts with.

Using the Swiss Model, we generated a homology model of our mutant protein with CTX-M-15 (PDB: 4HBT) as the template. The RMSD value of the model was found to be 0.09 Å by the structure assessment tool in the Swiss model website and 0.161 Å in PyMol. Visual inspection showed a high structure similarity when the model was superimposed over the template (PDB: 4HBT). The orientation of the Asn (135) in CTX-M-15 and the Asp (135) of the model does not show a significant difference (Fig. 3a). Likewise, the Ile (176) in CTX-M-15 and Phe (176) of the model show the same orientation (Fig. 3b). Since the RMSD values of the model were less than 0.5 Å, we used the generated model for further docking analysis. Before docking mecillinam with the model or the template (PDB: 4HBT), we identified the residues of CTX-M-15 (hereafter referred to as wild-type CTX or WT-CTX) that interact with avibactam in the crystal structure PDB: 4HBU using pymol. Avibactam interacts with Ser-73, Asn-107, Ser-133, Asn-135, Asn-173, Thr-238 and Ser-240. The residue numberings followed here are their positions in the fasta sequence of the Genpept-ID AEQ20893.1 (obtained from BLDB by Naas et al.^10^). We first checked if mecillinam interacted with the same residues of WT-CTX as avibactam via docking and found it to be interacting with the residues Ser73, Ser240, Lys237 (Fig. 4a) of the protein with a predicted lowest binding energy of ΔG = − 6.79 and with cluster as well as reference RMS values of 0 and 11.52 respectively. Besides Lys237, mecillinam was predicted to bind to Ser73 and Ser240 as avibactam in the crystal structure PDB: 4HBU. Then, we checked if mecillinam could also bind to these residues in the generated model via docking and found it to interact with Lys237, Ser240, and Asp-135 with the lowest binding energy of ΔG = − 5.99 (Fig. 4b). The cluster and reference RMS values are 0 and 11.54, respectively.

Previously, Rosenkilde et al.^21^ showed that N135 to D135 in CTX-M-15 causes resistance towards mecillinam in their in vitro experiments^21^. This was further validated by Nielsen et al.^20^, who observed the loss of sensitivity to pivmecillinam in a patient with UTI who developed a mutant CTX-M-15 with N135 to D135 mutation (also called CTX-M-127) harboring *E. coli* post-treatment with mecillinam^20^. These studies suggest that the N135 to D135 mutation could be responsible for the resistance towards mecillinam. Our predicted binding of the mecillinam to the D-135 residue of the model indicates that the binding to the mutated residue may have contributed toward mecillinam resistance. However, this should be further validated by co-crystallizing the mutant (or CTX-M-127) with mecillinam.

Ampicillin, co-trimoxazole, and nalidixic acid were introduced in Bangladesh in 1972, 1982, and 1985, respectively. Due to the appearance of resistance, Pivmecillinam was introduced a couple of years later. A few cases of mecillinam resistance were reported in 1991, which increased to around 50% in clinical *Shigella* spp. isolates in 1996^32^. In a recent meta-analysis, the prevalence of mecillinam resistance was measured at 13.7% (95% Confidence Interval 5.5–34.1) among 20,485 *Shigella* spp. clinical isolates^33^. Our work reports the occurrence of CTX-M-127 and has proven these evidences of mecillinam resistance in Bangladesh.

### Supplementary Information

Supplementary Information.

## Data Availability

The datasets used and/or analysed during the current study available from the corresponding author on reasonable request.

## References

[1] Okesola, A.O. & Makanjuola O. Resistance to third-generation Cephalosporins and other antibiotics by Enterobacteriaceae in Western Nigeria. *Am. J. Infect. Diseases*. **5**(1), 17–20 (2009).

[2] Knothe H, Shah P, Krcmery V, Antal M, Mitsuhashi S (1983). Transferable resistance to cefotaxime, cefoxitin, cefamandole and cefuroxime in clinical isolates of Klebsiella pneumoniae and Serratia marcescens. Infection..

[3] Philippon A, Labia R, Jacoby G (1989). Extended-spectrum beta-lactamases. Antimicrob. Agents Chemother..

[4] Bernard H, Tancrede C, Livrelli V, Morand A, Barthelemy M, Labia R (1992). A novel plasmid-mediated extended-spectrum beta-lactamase not derived from TEM- or SHV-type enzymes. J. Antimicrob. Chemother..

[5] Barthelemy M, Peduzzi J, Bernard H, Tancrede C, Labia R (1992). Close amino acid sequence relationship between the new plasmid-mediated extended-spectrum beta-lactamase MEN-1 and chromosomally encoded enzymes of Klebsiella oxytoca. Biochim. Biophys. Acta..

[6] Ishii Y, Ohno A, Taguchi H, Imajo S, Ishiguro M, Matsuzawa H (1995). Cloning and sequence of the gene encoding a cefotaxime-hydrolyzing class A beta-lactamase isolated from Escherichia coli. Antimicrob. Agents Chemother..

[7] Bauernfeind A, Stemplinger I, Jungwirth R, Ernst S, Casellas JM (1996). Sequences of beta-lactamase genes encoding CTX-M-1 (MEN-1) and CTX-M-2 and relationship of their amino acid sequences with those of other beta-lactamases. Antimicrob. Agents Chemother..

[8] Palucha A, Mikiewicz B, Hryniewicz W, Gniadkowski M (1999). Concurrent outbreaks of extended-spectrum beta-lactamase-producing organisms of the family Enterobacteriaceae in a Warsaw hospital. J. Antimicrob. Chemother..

[9] Poirel L, Kampfer P, Nordmann P (2002). Chromosome-encoded Ambler class A beta-lactamase of Kluyvera georgiana, a probable progenitor of a subgroup of CTX-M extended-spectrum beta-lactamases. Antimicrob. Agents Chemother..

[10] Naas T, Oueslati S, Bonnin RA, Dabos ML, Zavala A, Dortet L (2017). Beta-lactamase database (BLDB)—Structure and function. J. Enzyme Inhib. Med. Chem..

[11] Alemu A (2014). Microbial contamination of currency notes and coins in circulation: A potential public health hazard. Biomed. Biotechnol..

[12] Lamichhane, J., Adhikary, S., Gautam, P., Maharjan, R. & Dhakal, B. Risk of handling paper currency in circulation chances of potential bacterial transmittance. *Nepal J. Sci. Technol*. **10**, 161–166 (2010).

[13] Alwakeel S, Nasser L (2011). Bacterial and fungal contamination of Saudi Arabian paper currency and cell phones. Asian J. Biol. Sci..

[14] Basavarajappa KG, Rao PN, Suresh K (2005). Study of bacterial, fungal, and parasitic contamination of currency notes in circulation. Indian J. Pathol. Microbiol..

[15] Firoozeh F, Dadgostar E, Akbari H, Zibaei M, Sadjjadian SMS, Moshtaghi MM (2017). Bacterial contamination of iranian paper currency and their antibiotic resistance patterns. Int. J. Enteric Pathogens..

[16] Gedik H, Voss TA, Voss A (2013). Money and transmission of bacteria. Antimicrob. Resistance Infect. Control..

[17] Jalali S, Kohli S, Latka C, Bhatia S, Vellarikal SK, Sivasubbu S (2015). Screening currency notes for microbial pathogens and antibiotic resistance genes using a shotgun metagenomic approach. PLoS One..

[18] Ahmed SM, Naher N, Tune SNBK, Islam BZ (2022). The implementation of National Action Plan (NAP) on antimicrobial resistance (AMR) in Bangladesh: Challenges and lessons learned from a cross-sectional qualitative study. Antibiotics (Basel)..

[19] Ahmed I, Rabbi MB, Sultana S (2019). Antibiotic resistance in Bangladesh: A systematic review. Int. J. Infect. Diseases..

[20] Nielsen KL, Hansen KH, Nielsen JB, Knudsen JD, Schonning K, Frimodt-Moller N (2019). Mutational change of CTX-M-15 to CTX-M-127 resulting in mecillinam-resistant *Escherichia coli* during pivmecillinam treatment of a patient. Microbiol. Open..

[21] Rosenkilde CEH, Munck C, Porse A, Linkevicius M, Andersson DI, Sommer MOA (2019). Collateral sensitivity constrains resistance evolution of the CTX-M-15 Î^2^-lactamase. Nat. Commun..

[22] Waterhouse A, Bertoni M, Bienert S, Studer G, Tauriello G, Gumienny R (2018). SWISS-MODEL: Homology modeling of protein structures and complexes. Nucleic Acids Res..

[23] Morris, G.M., Huey, R., Olson, A.J. Using AutoDock for ligand-receptor docking. *Curr. Protoc. Bioinform*. Chapter 8:Unit 8 14 (2008). 10.1002/0471250953.bi0814s24 19085980

[24] Biemer JJ (1973). Antimicrobial susceptibility testing by the Kirby-Bauer disc diffusion method. Ann. Clin. Lab. Sci. (1971)..

[25] Pournaras S, Zarkotou O, Poulou A, Kristo I, Vrioni G, Themeli-Digalaki K (2013). A combined disk test for direct differentiation of carbapenemase-producing enterobacteriaceae in surveillance rectal swabs. J. Clin. Microbiol..

[26] Sanger F, Nicklen S, Coulson AR (1977). DNA sequencing with chain-terminating inhibitors. Proc. Natl. Acad. Sci. USA..

[27] Kumar S, Stecher G, Li M, Knyaz C, Tamura K (2018). MEGA X: Molecular evolutionary genetics analysis across computing platforms. Mol. Biol. Evol..

[28] De Lano WL (2002). Pymol: An open-source molecular graphics tool. CCP4 Newslett. Pro. Crystallogr..

[29] Khan AT, Lal M, Ray Bagdi P, Sidick Basha R, Saravanan P, Patra S (2012). Synthesis of tetra-substituted pyrroles, a potential phosphodiesterase 4B inhibitor, through nickel(II) chloride hexahydrate catalyzed one-pot four-component reaction. Tetrahedron Lett..

[30] Sushmita DL, Stefano M, Thomas D-R, Manuela B, De Filomena L, Gautam S (2013). Structural insight into potent broad-spectrum inhibition with reversible recyclization mechanism: Avibactam in complex with CTX-M-15 and Pseudomonas aeruginosa AmpC & #x3b2;-Lactamases. Antimicrob. Agents Chemother..

[31] Younes A, Hamouda A, Dave J, Amyes SG (2011). Prevalence of transferable blaCTX-M-15 from hospital- and community-acquired *Klebsiella pneumoniae* isolates in Scotland. J. Antimicrob. Chemother..

[32] Hossain MA, Rahman M, Ahmed Q, Malek M, Sack R, Albert M (1998). Increasing frequency of mecillinam-resistant shigella isolates in urban Dhaka and rural Matlab, Bangladesh: A 6 year observation. J. Antimicrob. Chemother..

[33] Ahmed S, Chowdhury MIH, Sultana S, Alam SS, Marzan M, Islam MA (2023). Prevalence of antibiotic-resistant Shigella spp in Bangladesh: A Systematic review and meta-analysis of 44,519 samples. Antibiotics (Basel)..

