# Supplementary material for: CTX-M-127 with I176F mutations found in bacteria isolates from Bangladeshi circulating banknotes
Source: Sci Rep. 2024 Mar 11;14:5866. doi: 10.1038/s41598-024-56207-x (PMC10928162; doi:10.1038/s41598-024-56207-x)
Supplement: Supplementary file 1 — Supplementary Information. [file 41598_2024_56207_MOESM1_ESM.docx]

**Supplementary Information**

Title:

CTX-M-127 with I176F mutations found in bacteria isolates from Bangladeshi circulating banknotes.

Contributors:

^1^Md. Zannat Ali

^2^Sankaranarayanan Srinivasan

^1^Selina Akter

Affiliation:

^1^Department of Microbiology, Jashore University of Science and Technology, Bangladesh

^2^Department of Biotechnology, Indian Institute of Technology Madras, India

Table S1: Primers used for PCR amplification of resistance gene.

| Primer | Sequence(5’­-3’) | Target Gene | Annealing Temp. | Fragment (bp) |
| --- | --- | --- | --- | --- |
| M-1F | CCGTTTCCGCTATTACAAACCGTTG | *bla_CTX- M-1_* |  |  |
| M-1R | GGCCCATGGTTAAAAAATCACTGC |  | 62°C | 944 |
| M-2F | ATGATGACTCACAGCATTCG | *bla_CTX-M-2_* | 55°C | 833 |
| M-2R | TCCCGACGGCTTTCCGCGTT |  |  |  |
| M-8F | TTTGCCCGTGCGATTGG | *bla_CTX- M-8_* | 53°C | 368 |
| M-8R | CGACTTTCTGCCTTCTGCTCT |  |  |  |
| M-9F | ATGGTGACAAAGAGAGTGCA | *bla_CTX- M-9_* | 55°C | 870 |
| M-9R | CCCTTCGGCGATGATTCTC |  |  |  |
| M-10F | GCAGCACCAGTAAAGTGATGG | *bla_CTX- M-10_* | 59°C | 524 |
| M-10R | GCGATATCGTTGGTGGTACC |  |  |  |
| M-14F | GAGAGTGCAACGGATGATG | *bla_CTX- M-14_* | 52°C | 941 |
| M-14R | TGCGGCTGGGTAAAATAG |  |  |  |
| TEM-F | TCAACATTTCCGTGTCG | *bla_TEM_* |  |  |
| TEM-R | CTGACAGTTACCAATGCTTA |  | 56°C | 860 |
| KPC-F | TGTCACTGTATCGCCGTCTAG | *bla_KPC_* |  |  |
| KPC-R | TTACTGCCCGTTGACGCCCAATCC |  | 60°C | 880 |
| NDM-F | TGCCCAATATTATGCACCCGG | *bla_NDM_* |  |  |
| NDM-R | CGAAACCCGGCATGTCGAGA |  | 60°C | 204 |

Table S2: List of identified Gram negative bacteria isolated from circulating banknotes of Bangladesh and antibiotic susceptibility pattern.

| Serial code | Isolate Identification code | Presumptive Bacteria | Ceftriaxone(30µg)  R≤23, I=23-26, S≥28mm | Cefotaxime(30µg)  R≤23, I=24-29, S≥30mm | Cefoperazone(30µg)  R≤23, I=24-26, S≥28mm | Ceftazidime(30µg)  R≤23, I=24-27, S≥28mm | Meropenem(10µg)  R≤19, I=20-26, S≥27mm | Imipenem(10µg)  R≤16, I=17-20, S≥21mm | Aztreonam(30µg)  R≤22, I=23-27, S≥28mm |
| --- | --- | --- | --- | --- | --- | --- | --- | --- | --- |
| 23 | MC10T5 MAC | *E. coli* | 0 | 0 | 0 | 0 | 0 | 0 | 0 |
| 24 | MC10T7 MAC | *E. coli* | 0 | 0 | 0 | 0 | 0 | 0 | 0 |
| 25 | MC20T2 MAC | *Pseudomonas spp* | 0 | 0 | 0 | 0 | 0 | 0 | 0 |
| 26 | MC50T1 MAC | *Pseudomonas spp* | 0 | 0 | 0 | 0 | 0 | 0 | 0 |
| 27 | M20T2 MAC | *Acinetobacter spp* | 0 | 0 | 0 | 0 | 0 | 0 | 0 |
| 28 | M10T8-a MAC | *Klebsiella spp* | 0 | 0 | 0 | 0 | 0 | 0 | 0 |
| 29 | M10T8-b MAC | *Pseudomonas spp* | 0 | 0 | 0 | 0 | 0 | 0 | 0 |
| 30 | M10T5 MAC | *E. coli* | 0 | 0 | 0 | 0 | 0 | 0 | 0 |
| 31 | M10T7-a MAC | *Pseudomonas spp* | 0 | 0 | 0 | 0 | 0 | 0 | 0 |
| 32 | M10T7-b MAC | *Acinetobacter spp* | 0 | 0 | 0 | 0 | 0 | 0 | 0 |
| 33 | M5T1 MAC | *Acinetobacter spp* | 0 | 0 | 0 | 0 | 0 | 0 | 0 |
| 34 | M50T2-a MAC | *E. coli* | 0 | 0 | 0 | 0 | 0 | 0 | 0 |
| 35 | M50T2-b MAC | *Acinetobacter spp* | 0 | 0 | 0 | 0 | 0 | 0 | 0 |
| 36 | M2T3 MAC | *Pseudomonas spp* | 0 | 0 | 0 | 0 | 0 | 0 | 0 |
| 37 | M10T8-c MAC | *E. coli* | 0 | 0 | 0 | 0 | 0 | 0 | 0 |
| 38 | M20T3 MAC | *Acinetobacter spp* | 0 | 0 | 0 | 0 | 0 | 0 | 0 |
| 39 | M10T6-a MAC | *E. coli* | 0 | 0 | 0 | 0 | 0 | 0 | 0 |
| 40 | M10T6-b MAC | *Pseudomonas spp* | 0 | 0 | 0 | 0 | 0 | 0 | 0 |
| 41 | C10T6-a MAC | *Pseudomonas spp* | 0 | 0 | 0 | 0 | 0 | 0 | 0 |
| 42 | C10T6-b MAC | *E. coli* | 0 | 0 | 0 | 0 | 0 | 0 | 0 |
| 43 | C20T1 MAC | *Acinetobacter spp* | 0 | 0 | 0 | 0 | 0 | 0 | 0 |
| 44 | C2T3 MAC | *E. coli* | 0 | 0 | 0 | 0 | 0 | 0 | 0 |
| 45 | C10T8-a MAC | *E. coli* | 0 | 0 | 0 | 0 | 0 | 0 | 0 |
| 46 | C10T8-b MAC | *Pseudomonas spp* | 0 | 0 | 0 | 0 | 0 | 0 | 0 |
| 47 | MC10T7 MAC | *Pseudomonas spp* | 0 | 0 | 0 | 0 | 0 | 0 | 0 |
| 48 | MC50T2 MAC | *E. coli* | 0 | 0 | 0 | 0 | 0 | 0 | 0 |
| 49 | MC10T8 MAC | *Klebsiella spp* | 0 | 0 | 0 | 0 | 0 | 0 | 0 |
| 50 | MC20T3 MAC | *Acinetobacter spp* | 0 | 0 | 0 | 0 | 0 | 0 | 0 |
| 51 | C10T8-c MAC | *E. coli* | 0 | 0 | 0 | 0 | 0 | 0 | 0 |
| 52 | C10T7-a MAC | *Klebsiella spp* | 0 | 0 | 0 | 0 | 0 | 0 | 0 |
| 53 | C10T7-b MAC | *Pseudomonas spp* | 0 | 0 | 0 | 0 | 0 | 0 | 0 |
| 54 | C20T3 MAC | *E. coli* | 0 | 0 | 0 | 0 | 0 | 0 | 0 |
| 55 | C20T2 MAC | *E. coli* | 0 | 0 | 0 | 0 | 0 | 0 | 0 |
| 56 | C50T2-a MAC | *Klebsiella spp* | 0 | 0 | 0 | 0 | 0 | 0 | 0 |
| 57 | C50T2-b MAC | *Acinetobacter spp* | 0 | 0 | 0 | 0 | 0 | 0 | 0 |
| 58 | C50T2-c MAC | *Pseudomonas spp* | 0 | 0 | 0 | 0 | 0 | 0 | 0 |
| 62 | C5T_6_ MAC | *Pseudomonas spp* | 0 | 0 | 0 | 0 | 0 | 0 | 0 |
| 66 | C10T_8_ MAC | *E. coli* | 0 | 0 | 0 | 0 | 0 | 0 | 0 |
| 68 | C20T_3_ MAC | *E. coli* | 0 | 0 | 10 mm | 0 | 0 | 0 | 0 |
| 74 | C2T_6_(1) MAC | *Pseudomonas spp* | 0 | 0 | 0 | 0 | 0 | 0 | 0 |
| 76 | C5T_4_ MAC | *Pseudomonas spp* | 0 | 0 | 0 | 0 | 0 | 0 | 0 |
| 83 | C20T_2_(1) MAC | *E. coli* | 0 | 0 | 0 | 0 | 0 | 0 | 0 |

Table S3: Presence of antibiotic resistant genes detected by polymerase chain reaction (PCR) in Gram negative bacteria isolated from circulating banknotes of Bangladesh.

| **Serial code** | **Isolate Identification code** | ***bla_CTX-M-1_*** | ***bla_CTX-M-2_*** | ***bla_CTX-M-8_*** | ***bla_CTX-M-9_*** | ***bla_CTX-M-10_*** | ***bla_CTX-M-14_*** | ***bla_TEM_*** | ***bla_KPC_*** | ***bla_NDM_*** |
| --- | --- | --- | --- | --- | --- | --- | --- | --- | --- | --- |
| 34 | M50T2-a MAC | (+)ve | (-)ve | (-)ve | (-)ve | (+)ve | (-)ve | (+)ve | (-)ve | (+)ve |
| 35 | M50T2-b MAC | (-)ve | (-)ve | (-)ve | (-)ve | (-)ve | (+)ve | (+)ve | (-)ve | (+)ve |
| 37 | M10T8-c MAC | (+)ve | (-)ve | (-)ve | (-)ve | (+)ve | (-)ve | (+)ve | (-)ve | (+)ve |
| 38 | M20T3 MAC | (+)ve | (-)ve | (-)ve | (-)ve | (+)ve | (+)ve | (+)ve | (-)ve | (+)ve |
| 39 | M10T6-a MAC | (-)ve | (-)ve | (-)ve | (-)ve | (-)ve | (+)ve | (+)ve | (-)ve | (+)ve |
| 42 | C10T6-b MAC | (-)ve | (-)ve | (-)ve | (-)ve | (-)ve | (+)ve | (+)ve | (-)ve | (-)ve |
| 44 | C2T3 MAC | (-)ve | (-)ve | (-)ve | (-)ve | (-)ve | (+)ve | (+)ve | (-)ve | (-)ve |
| 46 | C10T8-b MAC | (-)ve | (-)ve | (+)ve | (-)ve | (+)ve | (-)ve | (-)ve | (-)ve | (-)ve |
| 47 | MC10T7 MAC | (+)ve | (-)ve | (-)ve | (-)ve | (+)ve | (+)ve | (+)ve | (-)ve | (+)ve |
| 48 | MC50T2 MAC | (+)ve | (-)ve | (-)ve | (-)ve | (+)ve | (-)ve | (+)ve | (-)ve | (+)ve |
| 49 | MC10T8 MAC | (+)ve | (-)ve | (+)ve | (-)ve | (+)ve | (-)ve | (+)ve | (+)ve | (+)ve |
| 50 | MC20T3 MAC | (+)ve | (-)ve | (+)ve | (-)ve | (+)ve | (+)ve | (+)ve | (-)ve | (+)ve |
| 51 | C10T8-c MAC | (+)ve | (-)ve | (-)ve | (-)ve | (+)ve | (-)ve | (+)ve | (+)ve | (-)ve |
| 52 | C10T7-a MAC | (+)ve | (-)ve | (-)ve | (-)ve | (-)ve | (+)ve | (+)ve | (-)ve | (-)ve |
| 53 | C10T7-b MAC | (+)ve | (-)ve | (-)ve | (-)ve | (+)ve | (-)ve | (+)ve | (-)ve | (-)ve |
| 54 | C20T3 MAC | (+)ve | (-)ve | (-)ve | (-)ve | (+)ve | (+)ve | (+)ve | (-)ve | (-)ve |
| 55 | C20T2 MAC | (+)ve | (-)ve | (-)ve | (-)ve | (+)ve | (-)ve | (-)ve | (-)ve | (-)ve |
| 56 | C50T2-a MAC | (+)ve | (-)ve | (-)ve | (-)ve | (-)ve | (+)ve | (+)ve | (-)ve | (-)ve |
| 57 | C50T2-b MAC | (+)ve | (-)ve | (-)ve | (-)ve | (+)ve | (+)ve | (+)ve | (-)ve | (-)ve |
| 58 | C50T2-c MAC | (+)ve | (-)ve | (-)ve | (-)ve | (+)ve | (-)ve | (+)ve | (-)ve | (-)ve |
| 62 | C5T_6_ MAC | (-)ve | (-)ve | (-)ve | (-)ve | (-)ve | (+)ve | (-)ve | (-)ve | (-)ve |
| 66 | C10T_8_ MAC | (-)ve | (-)ve | (-)ve | (-)ve | (+)ve | (-)ve | (-)ve | (-)ve | (-)ve |
| 68 | C20T_3_ MAC | (-)ve | (-)ve | (-)ve | (-)ve | (+)ve | (+)ve | (-)ve | (-)ve | (-)ve |
| 74 | C2T_6_(1) MAC | (-)ve | (-)ve | (-)ve | (-)ve | (+)ve | (-)ve | (-)ve | (-)ve | (-)ve |
| 76 | C5T_4_ MAC | (-)ve | (-)ve | (-)ve | (-)ve | (+)ve | (-)ve | (-)ve | (-)ve | (-)ve |


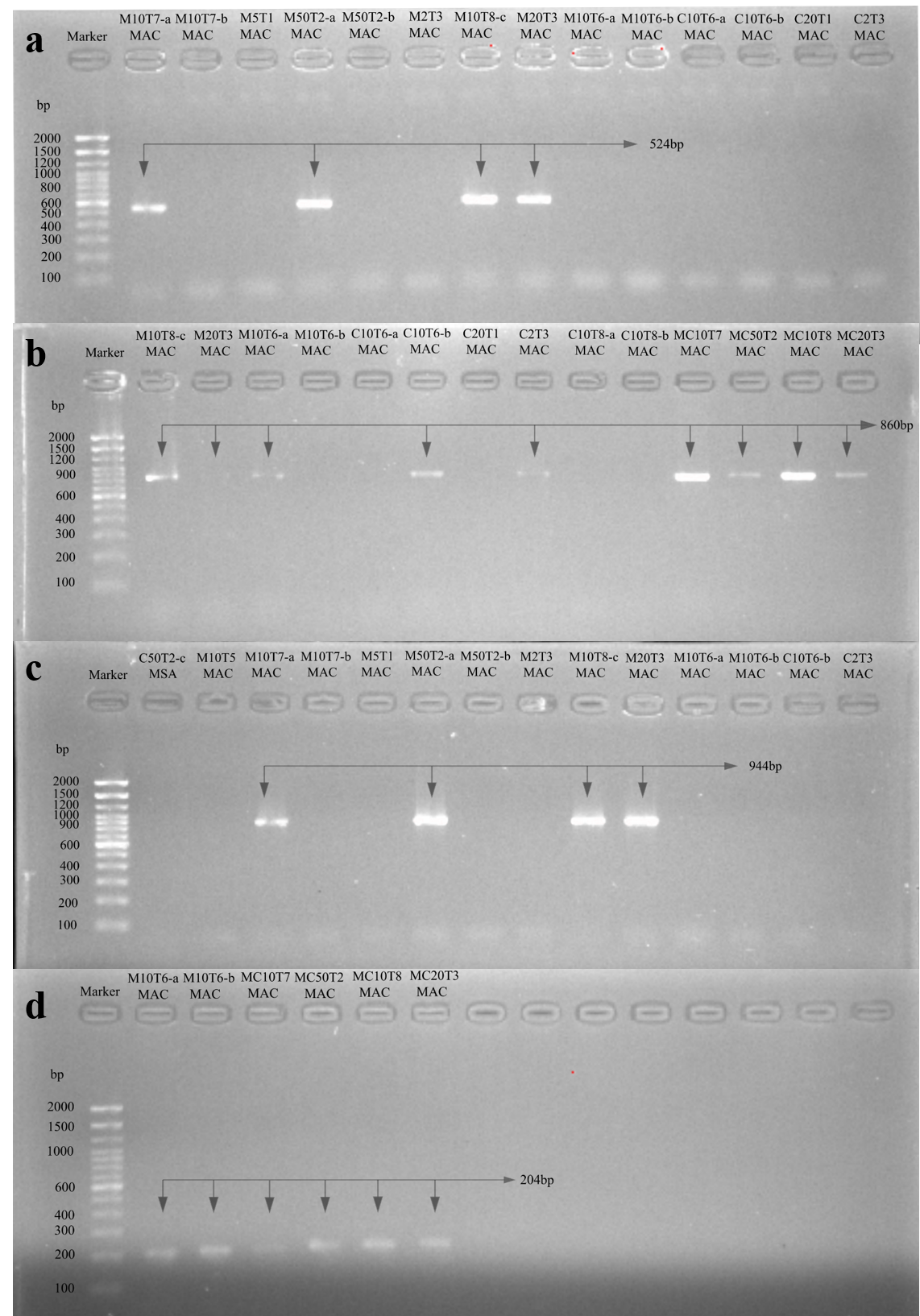


Figure S1: Representative agarose gel (1.5%) of PCR amplicon upon electrophoresis and staining with ethidium bromide; 524 bp amplicon of bla_CTX-M-10_ gene (A), 860 bp amplicon of bla_TEM_ gene (B), 944 bp amplicon of bla_CTX-M-1_ gene (C), and 204 bp amplicon of bla_NDM_ gene (D).

**.**


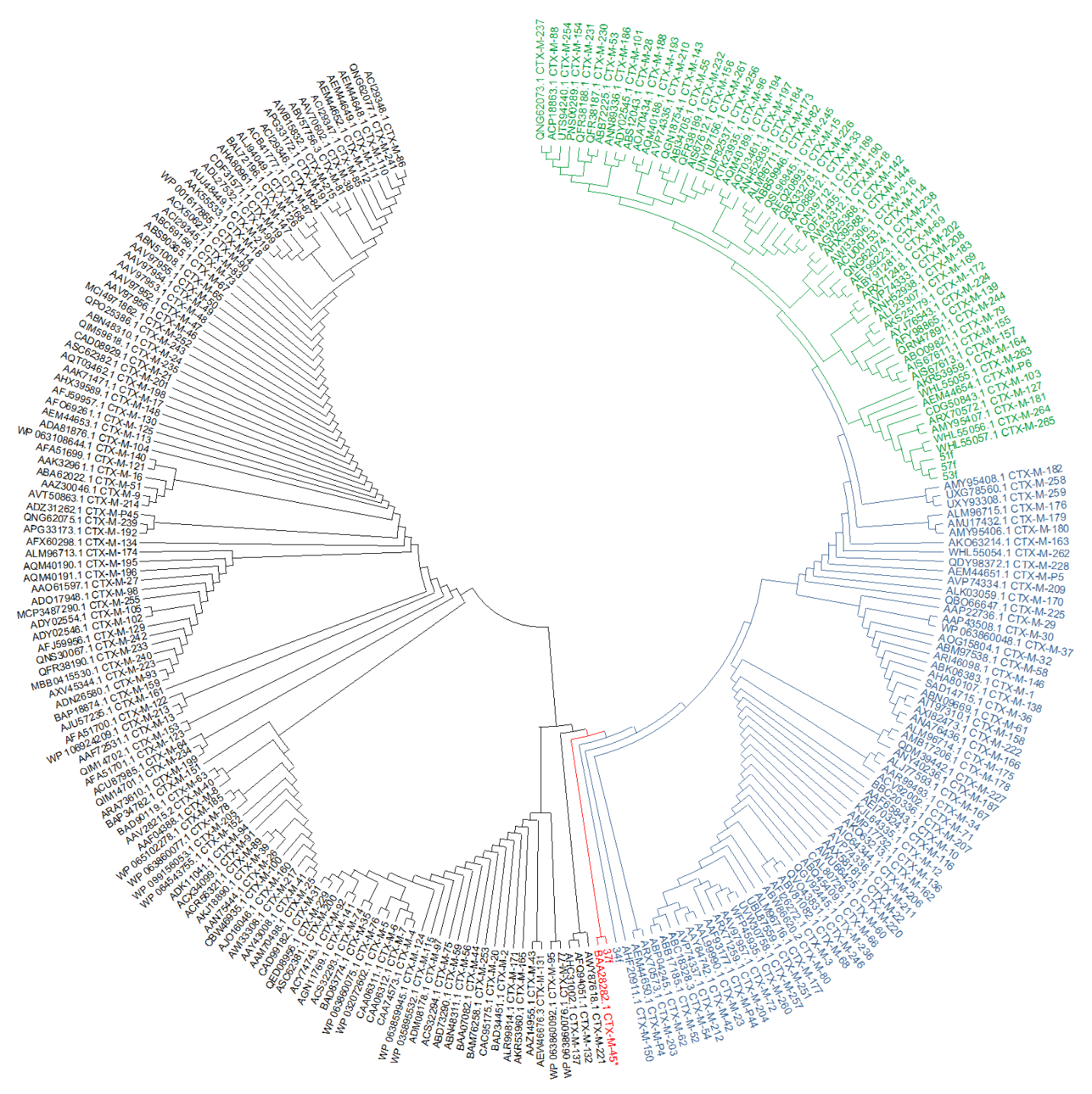


Figure S2: Phylogenetic tree built from the reference sequences for all available CTX-M (from beta-lactamase database, BLDB) and positioning sequences of this work to observe phylogenetic relationship.
